# A Hybrid Model for the Computationally-Efficient Simulation of the Cerebellar Granular Layer

**DOI:** 10.3389/fncom.2016.00030

**Published:** 2016-04-19

**Authors:** Anna Cattani, Sergio Solinas, Claudio Canuto

**Affiliations:** ^1^Laboratory of Neural Computation, Center for Neuroscience and Cognitive Systems @UniTn, Istituto Italiano di TecnologiaRovereto, Italy; ^2^Department of Brain and Behavioural Science, University of PaviaPavia, Italy; ^3^Department of Mathematical Sciences, Polytechnic University of TurinTorino, Italy

**Keywords:** neural networks, hybrid models, conductance-based models, continuum models, cerebellum

## Abstract

The aim of the present paper is to efficiently describe the membrane potential dynamics of neural populations formed by species having a high density difference in specific brain areas. We propose a hybrid model whose main ingredients are a conductance-based model (ODE system) and its continuous counterpart (PDE system) obtained through a limit process in which the number of neurons confined in a bounded region of the brain tissue is sent to infinity. Specifically, in the discrete model, each cell is described by a set of time-dependent variables, whereas in the continuum model, cells are grouped into populations that are described by a set of continuous variables. Communications between populations, which translate into interactions among the discrete and the continuous models, are the essence of the hybrid model we present here. The cerebellum and cerebellum-like structures show in their granular layer a large difference in the relative density of neuronal species making them a natural testing ground for our hybrid model. By reconstructing the ensemble activity of the cerebellar granular layer network and by comparing our results to a more realistic computational network, we demonstrate that our description of the network activity, even though it is not biophysically detailed, is still capable of reproducing salient features of neural network dynamics. Our modeling approach yields a significant computational cost reduction by increasing the simulation speed at least 270 times. The hybrid model reproduces interesting dynamics such as local microcircuit synchronization, traveling waves, center-surround, and time-windowing.

## 1. Introduction

Interesting phenomena in the brain often involve complex networks with an extremely large number of neurons. The description at the microscopic level of the whole network, i.e., the modeling of each single neuron and synapse, would lead to numerical models demanding prohibitive computational cost, even on the most advanced computers. The difficulties of such a description may be alleviated to some extent by identifying a hierarchy among interacting populations of neurons, and by using models with different resolutions and costs for simulating the behavior of different populations. Cell density may be a criterion to identify families of neurons and to partition the network in a multi-level manner, where each level corresponds to one or more species with comparable density. In the simplest situation of a two-level organization, this option leads to describe each neuron of the low-density population(s) by means of an ODE system, and to characterize the high-density population(s) by exploiting a PDE system that describes their ensemble as a continuum. The hybrid model collects the ODE and the PDE systems, as well as the fundamental interactions among them.

Several efforts have been made to understand and reproduce the activity of high-density populations by reducing the degrees of freedom from many, i.e., the variable states for each neuron, to few, and they have resulted in the formalization of different models. *Mean field, neural mass*, and *neural field models* are some of the results of various “passage to the continuum” approaches. A review concerning these models can be found in Bressloff (2012) and Deco et al. (2008). The major difference between neural-field models — such as the one we are going to present — and the others lies in the fact that the former account for the spatiotemporal evolution of the variables, rather than considering just their temporal evolution. A pillar formalization of a neural field model is proposed in Amari (1977) and Wilson and Cowan (1972, 1973), in which the macroscopic state variable is the mean firing rate. A more general neural-field model, not necessarily involving only firing rate variables, is presented in Touboul (2014).

We obtain a continuum model for the action potential of a dense population of neurons by starting from a discrete model and letting the number of neurons tend to infinity while keeping them confined in a bounded region. We identify limit operators, acting on the continuous variables, describing specific interactions: in particular, electrical couplings, like gap junctions or ephaptic coupling, are modeled in the limit by the Laplace differential operator, as has been rigorously justified in Canuto and Cattani (2014); on the contrary, chemical synaptic couplings produce non-local integral operators, i.e., spatial convolutions with suitable kernels (see e.g., Section 9.2 in Ermentrout and Terman, 2010). Once the expressions of both the discrete and the continuum model have been set, we describe in a fairly general form how the two models reciprocally interact, producing a hybrid model: in addition to terms in the equations describing interactions between “homogeneous” (i.e., discrete-discrete, or continuous-continuous) variables, new terms are added to account for the “heterogeneous” interactions (i.e., between discrete and continuous, or specialized continuous and discrete, variables).

To validate our new method in a complete workflow we applied it to a realistic computational problem, the neurodynamics of the cerebellar granular layer. Interest in the cerebellum dates back to the morphological studies carried out by Ramon y Cayal and Camillo Golgi, the electroencephalography studies carried out in Adrian (1935) and the motor impairment manifest in World War I and II patients with cerebellar lesions studied in Holmes (1917). Only later on, the cerebellum's fine structure inspired theories built to link network structure to function. This school produced a long stream of seminal papers (Braitenberg and Atwood, 1958; Marr, 1969; Albus, 1971; Ito, 1984), and initiated a line of research yet to be completed. Its peculiar structure comprehends series of highly regular, repeating units, each of which contains the same basic microcircuit. The similarity in repeating units, from architectural and physiological perspectives, implies that different regions perform similar computational operations on different inputs. These inputs originate from different parts of the brain, spinal cord, and sensory system which project directly into the cerebellum. In turn, the cerebellum projects to all motor systems. Despite the fact that the cerebellum's regularity has facilitated its description, it remains a network able to generate complex dynamics whose potential and function are yet not fully understood.

As underlined above, we specifically focus on the reconstruction of the cerebellar granular layer network (GLN). This network layer is densely populated by granule cells (GrCs) densely populated by granule cells (GrCs) and sparsely populated by Golgi cells (GoCs) providing an optimal application for our modeling approach. Our proposed hybrid model has been specialized to the description of the interactions between such populations. Interesting dynamics such as local microcircuit synchronization, center-surround and time-windowing, as already described in a previous and more biologically detailed model (Solinas et al., 2010b), are reproduced by the proposed model. Moreover, our model shows the emergence of traveling waves of network activity elicited by a generic input configuration of diffused activation of the GLN. Our hybrid model of the GLN is a very efficient computational representation of this network able to run large size (300, 000 neurons) simulations for a long simulated time (1 s) in about 1300 s on an ordinary laptop computer. The corresponding simulation for the biologically realistic model can only be run on a large computer cluster.

## 2. Materials and methods

### 2.1. The hybrid model

In this section we introduce the hybrid model by first showing how to model each individual neuron belonging to the same population. Here, intra-population communications are taken into account. Secondly, we perform a continuum limit of the discrete model that describes single neurons. This is motivated by the fact that, according to experimental evidence, even in a small brain area, the number of neurons is often huge. Finally, we present a hybrid model in which the discrete and the continuous models interact with each other.

Let us start by analyzing how to describe the dynamics of each individual neuron *i* in the network, where *i* = 1, ⋯*N* and *N* is the number of neurons in a population. To be precise, we consider three variables: the voltage-like variable *v*_*i*_, the recovery variable *r*_*i*_, and the variable *s*_*i*_ that describes the fraction of open channels in the synapses. In the most general case, each neuron is influenced by other neurons in the network by means of electrical and chemical connections, and its dynamics is also driven by basic principles of neural excitability. All these ingredients are taken into account in the following general model:

(1) $$\begin{array}{l} \begin{array}{cc} \frac{\text{d}v_{i}}{\text{d}t} & =f\left(v_{i},r_{i}\right)+I_{gap}^{i}+I_{syn}^{i},\\ \frac{\text{d}r_{i}}{\text{d}t} & =g\left(v_{i},r_{i}\right),\\ \frac{\text{d}s_{i}}{\text{d}t} & =\alpha_{i}\left(1-s_{i}\right)H_{\infty }\left(v_{i}-v_{T}\right)-\beta_{i}s_{i}, \end{array} \end{array}$$

where, $I_{\text{gap}}^{i}$ is the input current that accounts for electrical synapses, and $I_{\text{syn}}^{i}$ is that for chemical synapses. In particular,

(2) $$\begin{array}{l} \begin{array}{cc} I_{\text{gap}}^{i} & =d\underset{j\in Q\left(i\right)}{\sum }\left(v_{j}-v_{i}\right),\\ I_{\text{syn}}^{i} & =g_{\text{syn},\text{i}}\underset{j\in B\left(i\right)}{\sum }w_{ij}s_{j}\left(v_{i}-v_{\text{syn},\text{j}}\right), \end{array} \end{array}$$

where Q(*i*) and B(*i*), resp., collect the indexes of neurons connected to the *i*-th one by means of electrical and chemical synapses, resp., *w*_*ij*_ are positive weights describing the directed connection strength from *j* to *i*, *d* > 0 is the diffusion coefficient, *g*_*syn, *i**_ > 0 is the synaptic efficacy, and *v*_*syn, *j**_ is the reversal potential of the presynaptic neuron whose sign determines the synapse nature, either excitatory or inhibitory. In Destexhe et al. (1994), as well as Ermentrout and Terman (2010), a detailed classification of synaptic reversal potentials, linked to distinct neurotransmitter/receptor pairs is specified. Furthermore, among the wide variety of models which describe the basic properties of neural excitability, we select the FitzHugh-Nagumo model (FitzHugh, 1961):

(3) $$\begin{array}{l} \begin{array}{cc} f\left(v_{i},r_{i}\right) & =-v_{i}\left(a-v_{i}\right)\left(1-v_{i}\right)-r_{i},\\ g\left(v_{i},r_{i}\right) & =bv_{i}-cr_{i}, \end{array} \end{array}$$

which is phenomenologically derived from the biophysically-based Hodgkin-Huxley model. Here, *a, b, c* ∈ ℝ^+^ are parameters chosen so that *v*_*i*_ is a fast variable and *r*_*i*_ is a slow one. Finally, in the third equation in (1), α and β are positive parameters describing the forward and backward rate constants for transmitter binding, *v*_*T*_ is an *a priori* fixed threshold, and *H*_∞_ = *H*_∞_(*z*) is the Heaviside function such that *H*_∞_ = 0 if *z* < 0 and *H*_∞_ = 1 otherwise. Model (1) is supplemented by suitable initial conditions for the variables (*v*_*i*_, *r*_*i*_, *s*_*i*_).

In order to avoid prohibitive computational costs when the density of cells in a population is too high, we perform a “passage to the limit” as the number of neurons *N* tends to infinity in (1). In this way, we capture the dynamics of a neuronal population as a whole by describing three continuous variables *v*(*x, t*), *r*(*x, t*) and *s*(*x, t*) (having the same meaning as in (1)), where *x* is the spatial variable. Specifically, as *N* → ∞ in a fixed and bounded spatial region Ω ⊂ ℝ^*m*^, with *m* ∈ {1, 2, 3}, the discrete model (1) leads to the following integro-differential system of equations (hereafter, we simplify the notation by making the *t*-dependence of each variable implicit):

(4) $$\begin{array}{lll} \frac{\partial v\left(x\right)}{\partial t} & = & f\left(v\left(x\right),r\left(x\right)\right)+d^{*}\Delta v\left(x\right)\\ -g_{syn}\int_{R\left(x\right)}w\left(x,y\right)s\left(y\right)\left(v\left(x\right)-v_{syn}\left(y\right)\right)dy\\ \frac{\partial r\left(x\right)}{\partial t} & = & g\left(v\left(x\right),r\left(x\right)\right)\\ \frac{\partial s\left(x\right)}{\partial t} & = & \alpha \left(1-s\left(x\right)\right)H_{\infty }\left(v\left(x\right)-v_{T}\right)-\beta s\left(x\right), \end{array}$$

supplemented by boundary conditions for *v* and initial conditions for *v*, *r*, *s*. Here, d^*^ is the diffusion coefficient, *g*_*syn*_ > 0 is the synaptic efficacy, and R(*x*) denotes a region centered in *x*. The electrical synapse term, i.e., d^*^Δ*v*(*x*), is the result of two equivalent methods that lead to a non-trivial continuum limit, as shown in Canuto and Cattani (2014). On the other hand, the integral form of the chemical synapse term, i.e., $g_{syn}\left(\int_{R\left(x\right)}w\left(x,y\right)s\left(y\right)\left(v\left(x\right)-v_{syn}\left(y\right)\right)dy\right)$, is due to the fact that the set B(*i*) in (1) does not shrink to a point as *N* → ∞, as explained in Ermentrout and Terman (2010) and Cattani (2014). We refer to Cattani (2014) for a discussion on the mathematical well-posedness of this model. Hereafter, in order to distinguish between the discrete and continuous variables, we will denote the continuous variables by Greek letters.

As already mentioned in the Introduction, by comparing the cell densities we may diversify the description of the populations in the network. Specifically, this comparison determines if a population may be described by a set of discrete systems or by a continuous model. However, the key point is that neurons are linked to each other in a very intricate fashion depending on the brain areas. It follows that signal transmission among populations, in addition to intra-population connectivity, is an important feature to be taken into account to explore the emergent network dynamics. The essence of the hybrid model lies in the interaction coupling terms among different populations.

By, for simplicity, considering two populations only, on the one hand the set of cells in the low-density population is described by an ODE system:

(5) $$\begin{array}{l} \begin{array}{cc} \frac{\text{d}v_{i}}{\text{d}t} & =f\left(v_{i},r_{i}\right)+\phi \left(v_{i};v_{j},s_{j}\right)+\Phi \left(v_{i};\omega ,\sigma \right)+I_{ext}^{i},\\ \frac{\text{d}r_{i}}{\text{d}t} & =g\left(v_{i},r_{i}\right),\\ \frac{\text{d}s_{i}}{\text{d}t} & =\alpha_{i}\left(1-s_{i}\right)H_{\infty }\left(v_{i}-v_{T}\right)-\beta_{i}s_{i}, \end{array} \end{array}$$

where

(6) $$\begin{array}{l} \begin{array}{cc} \phi \left(v_{i};v_{j},s_{j}\right) & =d\underset{j\in Q\left(i\right)}{\sum }\left(v_{j}-v_{i}\right)-g_{syn}\underset{j\in B\left(i\right)}{\sum }w_{ij}s_{j}\left(v_{i}-v_{syn,\text{j}}\right) \end{array} \end{array}$$

takes into account inputs from other cells belonging to the same low-density population, whereas

(7) $$\begin{array}{l} \begin{array}{cc} \Phi \left(v_{i};\omega ,\sigma \right) & =\delta \Delta \omega \left(x_{i}\right)-\gamma_{syn}\int_{R_{i}}w\left(i,y\right)\sigma \left(y\right)\left(v_{i}-\omega_{syn}\left(y\right)\right)\text{d}y \end{array} \end{array}$$

models the signal transmission coming from the continuous population. Here, *x*_*i*_ indicates the spatial position of the neuron labeled by *i* from the discrete family, whereas R_*i*_ is the region occupied by the neurons from the continuous family whose synapses influence neuron *i*. The term $I_{ext}^{i}$ represents an external current coming from sources different from the two species here considered. On the other hand, the high-density population is characterized by a PDE system:

(8) $$\begin{array}{l} \begin{array}{cc} \frac{\partial \omega }{\partial t} & =F\left(\omega ,\rho \right)+\psi \left(\omega ,\sigma \right)+\text{Ψ}\left(\omega ;v,s\right)+I_{ext},\\ \frac{\partial \rho }{\partial t} & =G\left(\omega ,\rho \right),\\ \frac{\partial \sigma }{\partial t} & =\alpha \left(1-\sigma \right)H_{\infty }\left(\omega -\omega_{T}\right)-\beta \sigma , \end{array} \end{array}$$

where, similarly to (6),

(9) $$\begin{array}{lll} \psi \left(\omega ,\sigma \right)\left(\xi \right) & \;=\; & \delta \Delta \omega \left(\xi \right)\\ -\gamma_{syn}\int_{R\left(\xi \right)}w\left(\xi ,y\right)\sigma \left(y\right)\left(\omega \left(\xi \right)-\omega_{syn}\left(y\right)\right)\text{d}y \end{array}$$

describes interactions within the continuum population, while

(10) $$\begin{array}{lll} \text{Ψ}\left(\omega ;v,s\right)\left(\xi \right) & = & d\underset{j\in Q\left(\xi \right)}{\sum }\left(v_{j}-\omega \left(\xi \right)\right)\\ -g_{syn}\underset{j\in B\left(\xi \right)}{\sum }w\left(\xi ,j\right)s_{j}\left(\omega \left(\xi \right)-v_{syn,\text{j}}\right) \end{array}$$

describes the interactions between species, and I_*ext*_ = I_*ext*_(ξ) is an external current. We use the term *hybrid* to denote the mathematical model constituted by the coupled systems (5)–(7) and (8)–(10).

### 2.2. Application to the cerebellar granular layer network (GLN)

The formalization of the hybrid model developed above is suitable for describing a variety of networks in the brain characterized by a large difference in their population densities. This network feature is found in the cerebellum, and cerebellum-like structures (Bell et al., 2008) such as the dorsal cochlear nucleus in the auditory sytem, the dorsal octavolateral nucleus of the electrosensory system of the electrosensive fish, the electrosensory lobe of the mormyrid electric fish, the medial octavolateral nucleus of fish and amphibians that possess a lateral line system sensing pressure waves in water, the marginal layer of the optic tectum of ray-finned fishes and the rostrolateral nucleus of thalamus. In these systems a large population of small neurons project to a relatively small number of inhibitory units that either provide feedback to the first population, as cerebellar Golgi cells do, or forward the signal to downstream areas as cerebellar Purkinje cells do. The olfactory bulb and the striatum also present a similar disparity in their neuronal population and are suitable to be efficiently represented with our new method. Out of these examples the cerebellar cortex is the most extensively studied and modeled network. Its network structure can be abstracted following previous modeling work (Solinas et al., 2010a,b; Simões de Souza and De Schutter, 2011).

**Figure 1:**
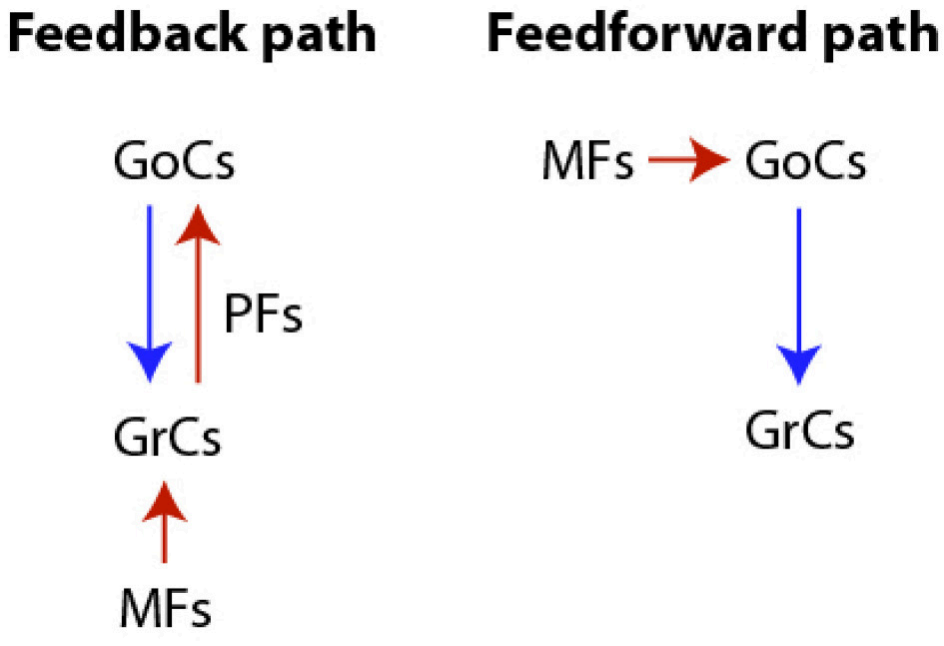
**Diagram of the cerebellar granular layer network pathways**. Both the feedback and the feedforward paths are characterized by excitatory (red arrow) and inhibitory (blue arrow) synapses.

We focus our study on reproducing the transformation imposed by the cerebellar granular layer network (GLN) to the input signals provided by the Mossy fibers (MFs). The GLN is composed of two main network pathways: a feedforward and a loop or feedback path, where both Granular cells (GrCs) and Golgi cells (GoCs) receive external excitatory inputs by MFs originating from the precerebellar nuclei neurons. MFs excite both cell populations duplicating their input into two pathways. Along the feedback path MFs synapse on GrCs. These excite GoCs through ascending axons and parallel fibers (PFs), and GoCs, in turn, inhibit GrCs. The second or feedforward path is constituted by the excitatory input from MFs to GoCs which terminates inhibiting GrCs. A diagram of both the feedback and feedforward pathways is shown in Figure 1.

**Figure 2:**
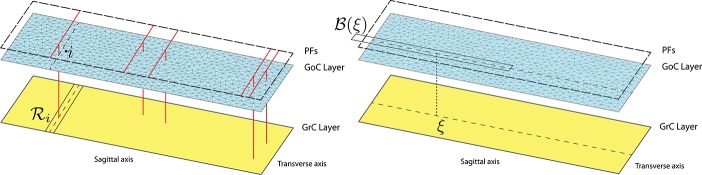
**Connection topology between GrCs and GoCs from a postsynaptic neuron perspective: GrCs linked to the ***i***-th GoC (left) and GoCs which are connected to the GrC at the point ξ (right)**. The domain decomposition of the GoC layer is obtained by exploiting the triangular mesh generator leading to a sparse mesh. Instead, a regular grid discretizes the GrC layer (not shown). The long and short edges of the rectangular map are 1500 and 500 μm, respectively.

Only a few cellular populations in the cerebellar cortex compose this geometrically regular network and are localized in three well distinct layers called *molecular, Purkinje*, and *granular*. The latter is densely populated by GrCs (density 4, 000, 000∕*mm*^3^) and sparsely by GoCs. The key point supporting the application of our new modeling method is that the number of GoCs significantly differs from that of GrCs: GoCs are very few compared to GrCs (Korbo et al., 1993; Solinas et al., 2010b; Billings et al., 2014) in the ratio of about 1 : 400. Thus, by virtue of this considerable density difference, there is clear motivation to study combined discrete and continuum models of the cerebellar granular layers. In particular, the variables (*v*_*i*_, *r*_*i*_, *s*_*i*_) describe each GoC through (5), while (ω, ρ, σ) portray the GrC species as a whole by means of (8). Inspired by assumptions in Simões de Souza and De Schutter (2011) and for modeling purposes, we consider the two populations belonging to two-dimensional parallel layers, as described in Figure 2. The bottom one consists of the GrC continuum and the upper one contains GoCs. A third layer, above them, consists of PFs.

We define our model topology and connectivity in the GLN taking into account the size and the fine structure of the biological network and describing, point by point, the corresponding structure in our network model. Specifically, we consider a region of the GLN with size 1500 μm along the sagittal axis, 500 μm on the transverse axis and 100 μm thick, including approximately 300, 000 GrCs, 750 GoCs, and 23, 000 MF terminals. However, in our representation the thickness of this flat volume is disregarded (Figure 2). The projection of MFs inside the GLN shows an abundant parasagittal branching. Each MF innervates multiple cerebellum lobules. Within the lobule, local branching gives rise to small clusters of about 8 MF terminals in a rectangular area of 200 μm along the transverse axis and 150 μm along the sagittal axis, data from the rat cerebellum (Sultan and Heck, 2003; Solinas et al., 2010b). About 50 GrCs, located within a sphere surrounding a MF terminal, project their dendrites (maximum length 30 μm, mean length 13.6 μm) onto the MF terminal. In this projection pattern the activation of a single MF gives rise to many small spots of activated GrCs with response intensity degrading from center to periphery (Mapelli et al., 2010b; Gandolfi et al., 2014). In our model, the GrC population is represented as a continuous sheet split into vertices by a regular tessellation allowing the calculation of numerical solutions. In this configuration, we assume that each MF terminal provides excitatory input to a subset of vertices located within 30 μm from the terminal and activated with an intensity decreasing with their distance, *x*, from the MF terminal as *sin*(*arccos*(*x*)) to mimic the flattening of the sphere. GoCs receive excitatory input from MF terminals from a wider area as GoC dendrites are longer than GrC dendrites and span a larger GLN volume (Dieudonné, 1998). Each GoC arborized axon reaches the granular layer throughout a parallelepiped volume (Barmack and Yakhnitsa, 2008) elongated along the sagittal direction, whose projection on the two-dimensional granular layer is a rectangle 650 μm long and 180 μm wide. A GoC sparsely inhibits GrCs lying inside the rectangle. Therefore, in our model, a GoC provides inhibitory input to a subset of the GrC nodes located within a rectangle elongated along the sagittal axis, sampling a total of 40 tessellation nodes in the rectangle. GrC axons, i.e., PFs, ascend to the molecular layer, bifurcate, and run parallel to each other in either direction along the transversal axis, our *x*-axis, for a few mm crossing the GoC apical dendrites. Each PF synapses onto many GoC dendrites along its path. The GoC apical dendrites branch out in all directions sampling PF input from a cylinder in the molecular layer represented in the original model by a circle of radius 50 μm (Dieudonné, 1998; Solinas et al., 2010b). In the hybrid model, each GoC is influenced by all the GrC nodes in a rectangle elongated along the transverse axis, covering the entire GLN extension, and narrow along the sagittal axis, covering 50 μm on either side of the PF wide stripe of the GLN (see Figure 2, left) accounting for the parasagittal extension of GoC dendrites (Solinas et al., 2010b). Notably, GoCs receive chemical excitatory synapses by GrCs. Furthermore, GoCs are linked among each other by gap junctions connecting their apical dendrites (Vervaeke et al., 2010). This electrical coupling is represented in our model by a diffusion term, *d* in (11), between the vertices of the discrete model, i.e., in a first approximation a GoC is coupled only with its nearest neighbors.

As already mentioned above, the Golgi cell system can be described by model (5). Due to the specific synapses that involve the GoCs as postsynaptic target, the general expressions of the functions ϕ and Φ, given in (6) and (7), take the following specific form:

(11) $$\begin{array}{l} \begin{array}{cc} \phi \left(v_{i};v_{j},s_{j}\right) & =d\underset{j\in Q\left(i\right)}{\sum }\left(v_{j}-v_{i}\right)\\ \Phi \left(v_{i};\omega ,\sigma \right) & =-\gamma_{syn}\int_{R_{i}}w\left(i,y\right)\sigma \left(y\right)\left(v_{i}-\omega_{syn}\right)\text{d}y. \end{array} \end{array}$$

Moreover, $I_{ext}^{i}=I_{mossy}^{i}$ is the excitatory input due to the MFs. Let us recall that, in (11), the reversal potential ω_*syn*_ may depend upon the specific type of synapse characterized by the nature of the presynaptic neuron and, thus, it must be included in the integral term. However, since here only GrCs influence GoCs by means of excitatory chemical synapses, we suppose ω_*syn*_ to be constant and we bring it out of the integral, obtaining

$$\begin{array}{l} \Phi \left(v_{i};\omega ,\sigma \right)=-\gamma_{syn}\left(\int_{R_{i}}w\left(i,y\right)\sigma \left(y\right)\text{d}y\right)\left(v_{i}-\omega_{syn}\right). \end{array}$$

The set R_*i*_ determines the area containing those GrCs which synapse onto the *i*-th Golgi cell. Taking into account that GrCs excite GoCs through the PFs, as specified above, we consider R_*i*_ as a thin rectangle whose horizontal symmetry axis is determined by the *i*-th cell projection (see Figure 2, left) having parasagittal extension of 50 μm.

Furthermore, the GrC continuum is described by the model (8), where the functions ψ and Ψ, introduced in (9) and (10), take the following specific form:

(12) $$\begin{array}{l} \begin{array}{cc} \psi \left(\omega ,\sigma \right)\left(\xi \right) & =\delta \Delta \omega \left(\xi \right),\\ \text{Ψ}\left(\omega ;v,s\right)\left(\xi \right) & =-g_{syn}\left(\underset{j\in B\left(\xi \right)}{\sum }w\left(\xi ,j\right)s_{j}\right)\left(\omega \left(\xi \right)-v_{syn}\right). \end{array} \end{array}$$

As above, the reversal potential *v*_*syn*_ of the GoC to GrC synapses is constant and it therefore does not contribute to the sum. The second equation accounts for the inhibition provided to the GrCs by the GoCs, while the first equation resulted from the passage to the limit of the high density neuronal population. Specifically, it introduces a reciprocal coupling of adjacent neurons as a result of local electric fields. This kind of interaction, named ephaptic coupling, has been documented in brain tissues (Bokil et al., 2001; Anastassiou et al., 2011), and cannot yet be excluded in the nature of the cerebellar granular layer tissues. In order to consider inputs from Mossy Fibers, we set I_*ext*_ = I_*mossy*_. The discrete set B(ξ) collects the indexes of GoCs which influence the GrC continuum at the point ξ, thus describing the connection topology. According to Barmack and Yakhnitsa (2008), a GoC axon reaches a rectangular region in the granular layer, centered on its soma; therefore, a possible choice is:

(13) $$\begin{array}{l} B\left(\xi \right):=\left\{j\in \mathbb{N}:x_{j}\in R_{\xi }\right\}, \end{array}$$

where *R*_ξ_ denotes such a rectangle centered on the projection of ξ on the GoC plane and oriented perpendicularly to the R_*i*_ direction (see Figure 2, right).

Since cells are described by the FitzHugh-Nagumo model, it is important to recall that the threshold is not involved in the single-neuron dynamics but it concerns presynaptic neurons at the synapse level. Indeed, when the presynaptic neuron exceeds the threshold, i.e., *v*_*T*_ and ω_*T*_, neurotransmitter release starts and influences the postsynaptic cells. Notably, *s* and σ are strictly positive functions of time (see Equations 5 and 8) and represent the contribution of a neuron's activity to its postsynaptic targets. In our model, a GrC-high-density-population node encodes its output in the dynamic variable σ that is used in the postsynaptic neuron to compute the synaptic current. Specifically, σ is incremented if in the node ω is larger that the threshold ω_*T*_, otherwise it decays exponentially toward zero with time constant 1∕(α + β) = 1 *ms* (α = 0.9 *ms*^−1^, β = 0.1 *ms*^−1^ Equation 8). This yields a rough approximation of the time profile of AMPA, NMDA, and KINATE current injected by a GrC synapse in the GoC neuron (cf. orange trace PF → GoC in Figure 3 of Solinas et al., 2010b). As a single point in the high-density-population represents more than one neuron, ω describes mean membrane potential and σ represents thresholded and filtered transmission to their postsynaptic targets. Therefore, σ can be seen as an approximation of the mean activity of the GrCs represented in a point of the continuous model. The same encoding holds also for the nodes of the GoC grid, each node represents one GoC, *v* is the neuron membrane potential and *s* represents the effect of the recent GoC activity on the postsynaptic GrCs.

We close this section with a few words about the numerical treatment of our model. Concerning GoCs — which form a discrete set — they are placed at the vertices of a quasi-uniform triangulation of the upper rectangular domain; we use the triangular mesh generator BBTR, described in Barbera and Berrone (2008), with the mesh refinement parameter chosen to yield 755 vertices (RefiningOptions parameter set to 0.00337; Figure 2). On the other hand, GrCs — which form a continuum in our model — are described by a set of partial differential equations that need to be discretized in space. To this end, we resort to a classical second-order centered finite difference method (see e.g., Quarteroni et al., 2000). In particular, we consider 23232 nodes in the domain, lying on a regular grid, to represent the 300, 000 GrCs. Therefore, using this grid size each vertex represents 13 GrCs. However, the results of the simulations turn out to be nearly independent on the GrCs grid refinement, as will be documented at the end of Section 3.2. Finally, time integration of the resulting coupled system of ordinary differential equations is accomplished by the MATLAB routine ODE45. We remark that the spatial discretization might be accomplished by finite elements instead of finite differences, thus allowing for the use of modern adaptive strategies, providing unstructured grids that adapt themselves to the formation of localized patterns; this will be object of future work. The MATLAB code used to run the GLN model is available on GitHub at the link: https://github.com/annacatt/HybridModel_CerebellarGranularLayer.git and it will made also available on Model DB (Migliore et al., 2003; Hines et al., 2007; Gleeson et al., 2014).

## 3. Results

Numerical simulations were performed with a two-fold aim: to show the dynamics that the hybrid model, composed of (5)–(11) and (8)–(12), is able to exhibit and to validate its capability to reproduce the GLN activity simulated in a biologically realistic model (Solinas et al., 2010b). In particular, in the validation step the values of the diffusion in the discrete and the continuous models, *d* and δ resp., were set to zero in agreement with the configuration of the biologically realistic model, where both the gap-junctions among GoCs and the ephaptic coupling among GrCs were not included.

### 3.1. Oscillatory activity in the granular layer

To present a sample dynamics of the hybrid model, we considered a network whose size is equivalent to a box with 1500 μm by 500 μm edges along the sagittal and transverse axes, and 100 μm thickness containing the cubic volume of brain tissue simulated in Solinas et al. (2010b). The parameter values of the FitzHugh-Nagumo model (3) were set to *a* = 0.25, *b* = 0.001, and *c* = 0.003. The synaptic reversal potentials in (11) and (12), resp., were set to ω_*syn*_ = 0.93 and *v*_*syn*_ = −0.2, resp. Moreover, using parameters of the same order of magnitude as in Simões de Souza and De Schutter (2011) and Solinas et al. (2010b), we fixed:

(14) $$\begin{array}{l} g_{syn}=0.8,\;d=0.005,\;I_{mossy}^{i}=I_{mossy}^{GoC}=0.1, \end{array}$$

for the Golgi cell discrete model, and

(15) $$\begin{array}{l} \gamma_{syn}=0.1,\;\delta =0.005,\;I_{mossy}\left(\xi \right)=I_{mossy}^{GrC}=0.2, \end{array}$$

for the Granular cell continuous one. In particular, $I_{mossy}^{GrC}$ was applied to 3% of MF, randomly chosen with uniform distribution, that excite the GrC nodes as described in Methods. Since in the real GLN GoCs also receive excitatory input from MFs, we assumed that 3% of GoCs randomly chosen with uniform distribution receive a non-zero $I_{mossy}^{GoC}$. This current was applied to GoCs for all *t* > 10 ms. MF current was maintained active to GrCs from *t* > 0 ms. The thresholds *v*_*T*_ and ω_*T*_ for GoCs and GrCs were both set to 0.5.

**Figure 3:**
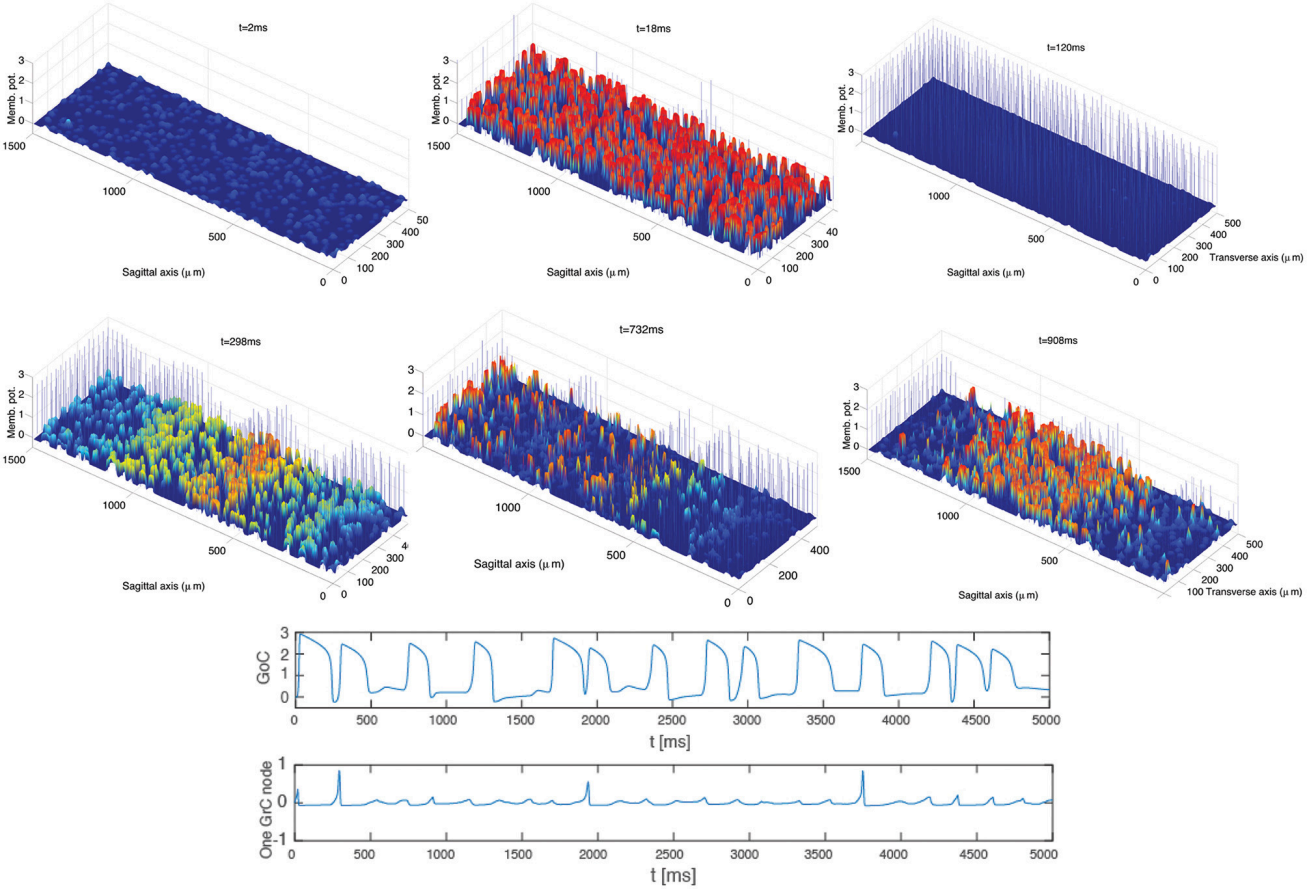
**Ensemble dynamics in the hybrid model**. After an initial period of initialization, a synchronous phenomenon within each population arises and the network activity shows oscillations with a frequency of 13 Hz. After a few cycles (*t* > 350 ms) a traveling wave phenomenon arises. The oscillatory frequency is unaffected by the spontaneous emergence of the waves. The waves of network activity diffuse along the sagittal axis. GrCs are represented with the colored continuous graph; GoCs are described with bars showing potentials multiplied by a factor 3 for graphical reasons. The bottom panels describe the dynamics of a sample GoC cell and GrC node up to 5 s. Both of them show a quasi-periodic dynamics.

Frames extracted from a video (see Supplementary Material: Video 1) of the GoC-GrC dynamics are shown in Figure 3. This dynamics was obtained by exploiting (5)–(11) and (8)–(12), assuming the topology described by (13). The excitatory input delivered by MFs to GrCs drives their activity above threshold and induces an increase in GoC potentials. The subsequent inhibition elicited in GrCs by the GoC inhibitory feedback loop (MFs-GrCs-PFs-GoCs-GrCs, see Figure 1) suppresses the GrC activity and the cycle restarts. In the early stages of the dynamics local microcircuit synchronous phenomena arise. The same phenomena arise in the biologically realistic model of reference (Solinas et al., 2010b) and this is a characteristic dynamics observed in the GLN *in vivo* (Vos et al., 1999) and computational models (Maex and DeSchutter, 1998). Furthermore, at a later time in the simulation, *t* > 350 ms, the synchronous dynamics spontaneously converts to an interesting dynamics where excitatory waves travel in the whole domain involving both GoCs and GrCs. In particular, these oscillations exhibit a quasi-periodic behavior at the level of both the network and the single node dynamics. The amplitude of the MF input can modulate the frequency of oscillations driving it into the θ or to higher β ranges. Moreover, the waves traveling along the sagittal axis show the high degree of synchrony of the GoCs aligned along the transversal axis that was shown in Vos et al. (1999), see Section 4.

### 3.2. Validation of the model: Center-surround and time-windowing

Over recent years several studies on the GoCs-GrCs network have been focused on the analysis of the integration of excitatory and inhibitory input by GrCs (Mapelli et al., 2010a,b; Solinas et al., 2010b; Gandolfi et al., 2014; Nieus et al., 2014). To validate our modeling reconstruction we focused on reproducing the spatial and temporal interaction of excitation and inhibition in the GLN following the work presented in Solinas et al. (2010b). This choice is motivated by the fact that neither gap-junctions among GoCs nor ephaptic coupling in the GrC population are considered in the biophysically realistic model (Solinas et al., 2010b). Indeed, significant comparisons with a previously published GLN model are here presented to show that our hybrid model produces a dynamics effectively expected for this network (Solinas et al., 2010b) in agreement with known publications in the field (Maex and DeSchutter, 1998; Santamaria et al., 2007; Dugué et al., 2009; Vervaeke et al., 2010).

According to Gandolfi et al. (2014) and Solinas et al. (2010b), the input delivered by a small bundle of MFs in the GLN elicits the activation of a cluster of GrCs, a spot 33 ± 5μm wide at 70% of the peak amplitude (Mapelli et al., 2010a). The spot is limited in size and in time by the properties of the feed-forward and feed-back inhibitory loops, due to the GoC integration properties and the arrangement of their axons. These phenomena, defined *center-surround* and *time-windowing* in D'Angelo and De Zeeuw (2009), are the result of the mismatch between the small area excited by the MFs and the wider area inhibited by GoCs activated directly and indirectly, through GrCs by the same MFs, in combination with the inherent delay of the inhibitory loops.

This section is devoted to present noticeable center-surround and time-windowing phenomena reproduced by models (5)–(11) and (8)–(12), where *d* and δ were set to zero. Differently from the previous section, now we set $I_{mossy}\left(\xi \right)=I_{mossy}^{GrC}=0.4$ in the granular cell layer to mimic the strong 2 spikes at 500 Hz used in Solinas et al. (2010b).

**Figure 4:**
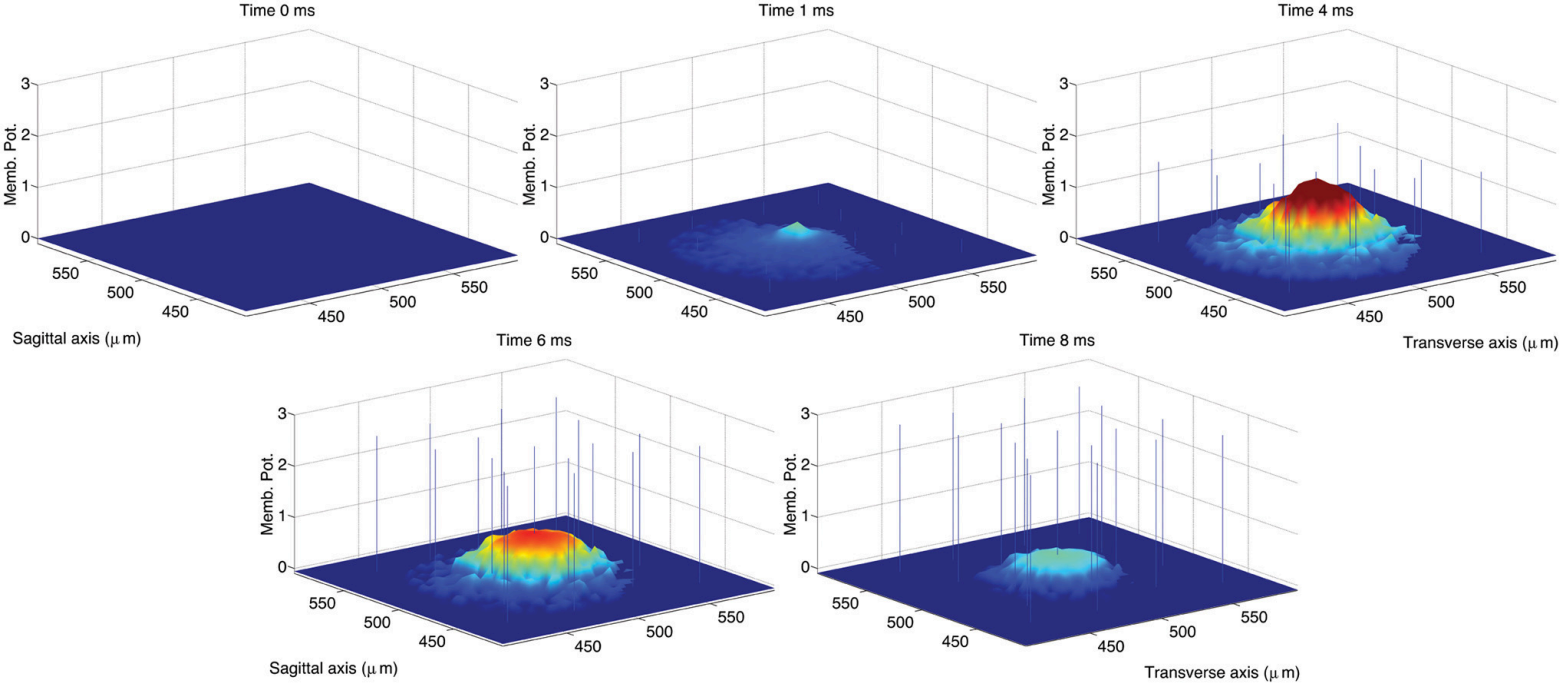
**Snapshots describing the center-surround phenomenon**. GoCs are excited by GrCs through the PFs. In turn, each active GoC inhibits GrCs lying on a thin rectangle. The maximal activation is reached at *t* = 4 ms and the diameter of the spot at 70% of the maximum amplitude is 36 μm. The stimulus is set on at *t* = 0 ms and set off at *t* = 5 ms.

The activation of a spot in the network center was achieved in the original model by activating the 8 MF terminals located within a sphere of radius equal to 20 μm located in the network center. Considering that the average length of GrC dendrites was set to 14 μm, the resulting excited volume was a sphere of radius equal to about 34 μm. In the simulations we ran to reproduce the impulse response of the GLN, we mimicked this activation by providing excitatory input to GrC vertices within a circle with radius equal to 34 μm located in the network center. We first ran a control simulation reproducing a spot of activation in the network center of the same size of the spot obtained in the original model (Solinas et al., 2010b; data not shown). In a second simulation, we increased the radius of the activated area to 70 μm in order to achieve a spot 33 μ*m* wide at 70% of the maximum peak amplitude (Mapelli et al., 2010a) as shown in Figure 4.

**Figure 5:**
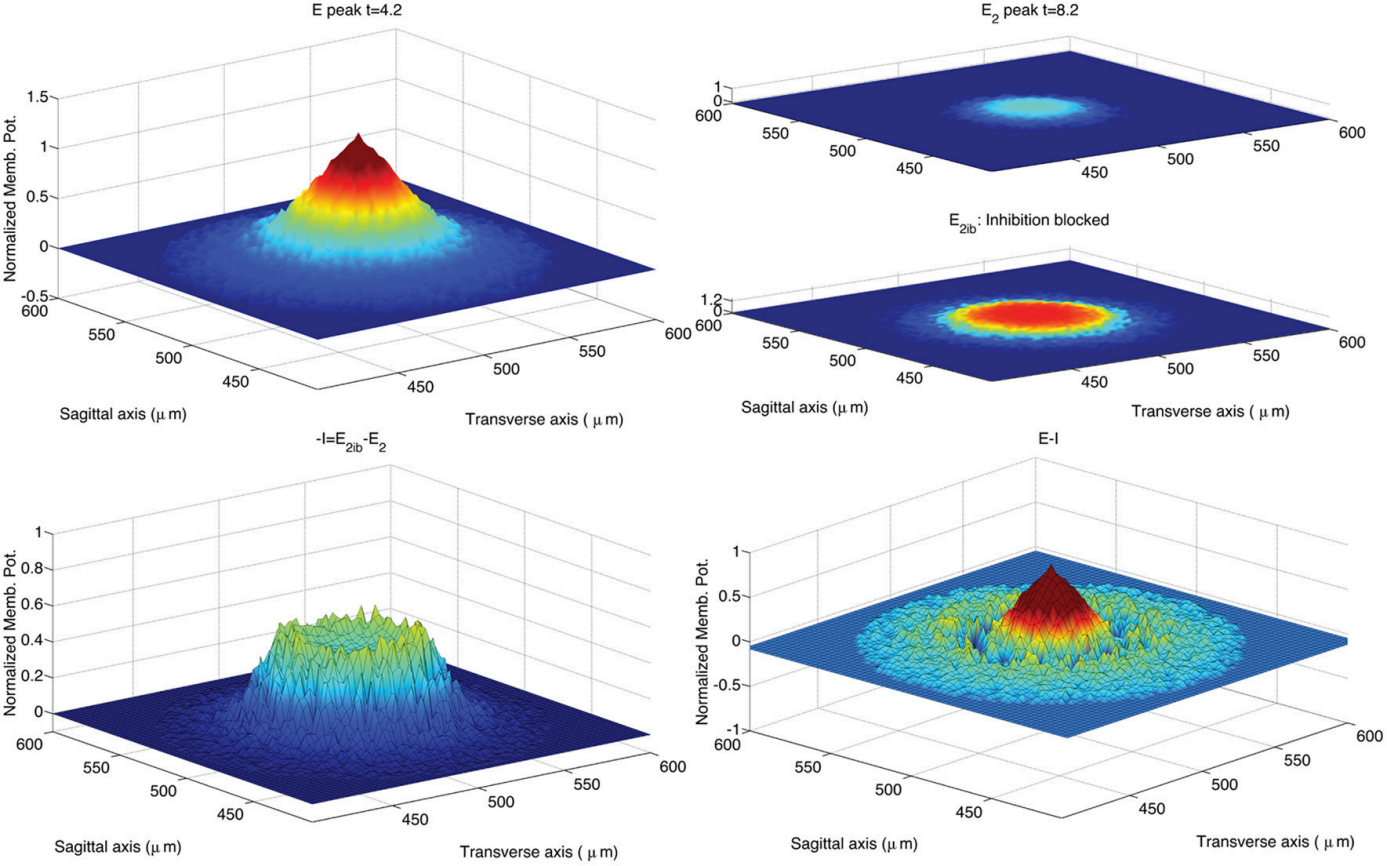
**Response of the hybrid model to 5 ms pulse delivered by MFs to GrCs and GoCs**. The GLN activation at time 4.2 ms from the initiation of the stimulus shows the maximal activation yielded by the excitatory input (*E* peak; upper left panel). After 4 ms the GLN activation fades off due to the emergence of the inhibitory feedback (*E*_2_ peak; upper right panel). The time of the *E*_2_ peak is chosen at 4.2 ms after the peak response to reproduce a data analysis following the methods used in Solinas et al. (2010b). Partial block of inhibitory synapses increases the *E*_2_ peak amplitude and spatial extension (*E*_2*ib*_; upper right panel). The amount of inhibition is calculated as the change in GLN activation amplitude due to the partial block of inhibition at 8.2 ms from the stimulus initiation (−*I*; lower left panel). The center-surround is represented as in Solinas et al. (2010b) as the difference from the *E* peak and the inhibition *I* (lower right panel). The stimulus is carried by all the MFs included in a circle located in the network center (250, 250)μ*m* and with radius equal to 70μ*m*. The intensity of stimulation is decreased from center to periphery of the circle following an exponential profile (intensity from 0.4 to 0 with space constant 0.6). The input intensity is randomized using an additive random noise with range [0, 0.05]. The so activated spot (upper left panel) has a diameter of 36μ*m* if measured at 70% of its peak amplitude (Mapelli et al., 2010b). The CPU time required to run this simulation was 2 s on a Apple® MacBook Pro (Intel Core 2 Duo 2.93 GHz).

Figure 5 shows the GLN response to a stimulus set on at *t* = 0 ms and set off at *t* = 5 ms. The spot size increases over time also after the end of the stimulus. According to Solinas et al. (2010b), the center-surround organization of the inhibitory projections shapes the GLN response in space and in time. In order to highlight the effect of inhibition, Figure 5 compares the spot of Figure 4 with the spot achieved after partial block of the inhibitory GoC to GrC synapses and synaptic strength reduced from 0.1 to 0.03. More precisely, we reproduce in Figure 5 the same computational steps taken in Solinas et al. (2010b). After the onset of the MF input the GLN initiates its response with 1 ms of delay, reaching its maximal activation after 4 ms indicated as *E* peak in Figure 5. After 8 ms the GLN activation fades off due to the emergence of the inhibitory feedback and we chose this time to measure the *E*_2_ peak. After partial block of the inhibitory synapses, the *E*_2_ peak increases in amplitude and extension (inhibition partially blocked: *E*_2*ib*_). As in Solinas et al. (2010b), the amount of inhibition *I* is calculated as the change in GLN activity amplitude due to the partial block of inhibition. Like in Solinas et al. (2010b), the difference between the *E* peak and the inhibition *I* reveals, in the lower right panel of Figure 5, the center-surround organization of inhibition as the central peak surrounded by a deeper crown.

**Figure 6:**
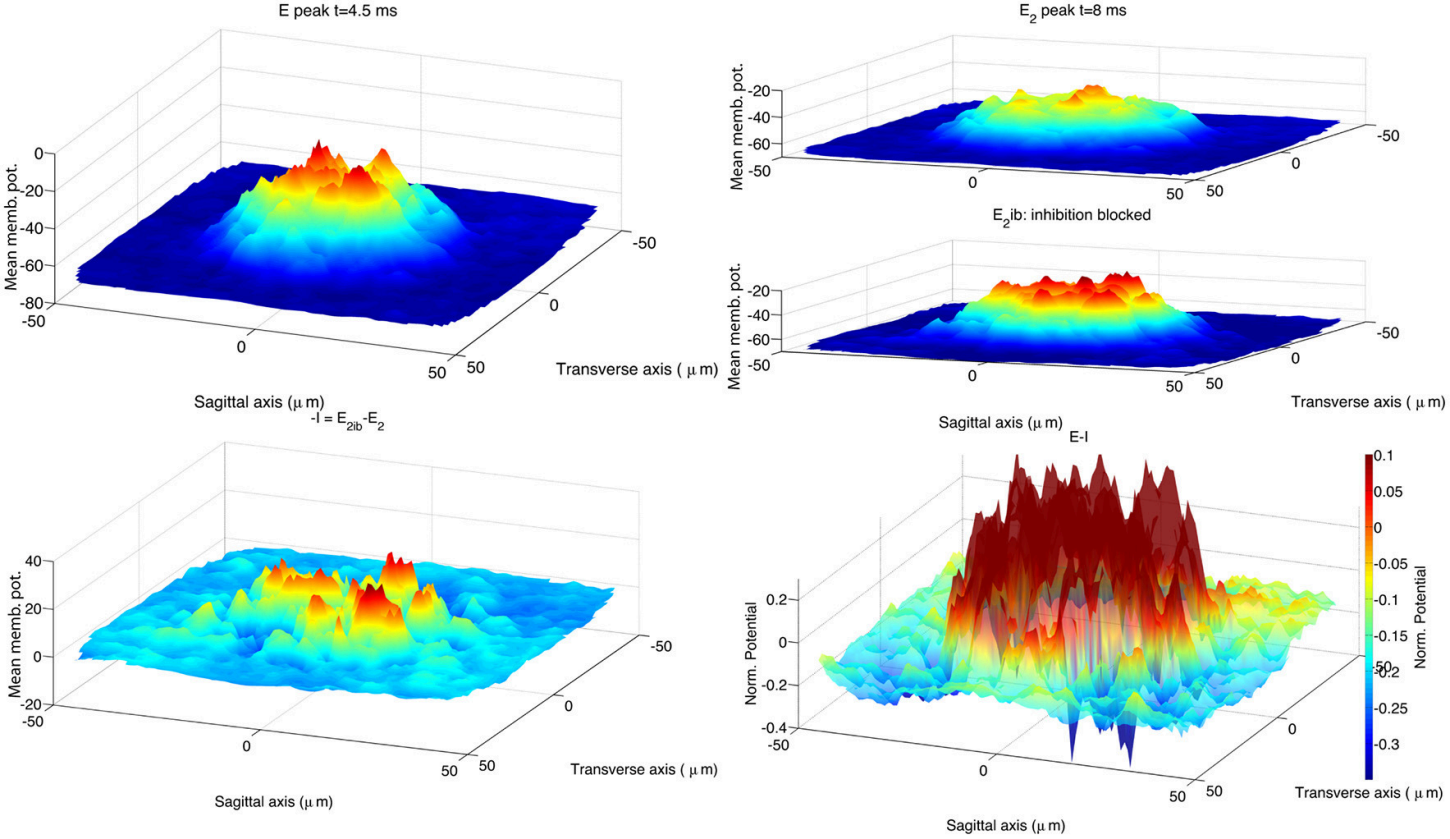
**Response of the biologically realistic model (Solinas et al., 2010b) to the activation of 13 MF terminals located within 20 μm from the network center**. As in the 2010 publication, at the end of each simulation the voltage trace of GrCs aligned along the vertical axis are pooled together to represent their ensemble activity. The resulting 2*D* surface represents the activity of this 3*D* GLN network (vertical axes show the mean membrane potential in mV). The simulations in control and partial inhibition block configuration are repeated 10 times, using each time a different network structure. The 2*D* surfaces belonging to each configuration class are used to build an average response for that class of GLN to the stimulus. The arrangement of data plots replicates the organization of Figure 5. The activated spot (upper left panel) has a diameter of 36μ*m* if measured at 70% of its peak amplitude (Mapelli et al., 2010b). The stimulus consists in a sequence of 3 spikes with an inter-spike interval of 333 ms. The first peak of GLN activation is reached within 4.5 ms from stimulus onset (*E* peak; upper left panel). The GLN shows a second peak of activity *E*_2_ with latency 8.2 ms from stimulus onset (upper right panel). Blocking inhibitory synapses induces a generalized increase of the GLN activity from this time on, note the *E*_2_ peak amplitude and its spatial extension (*E*_2*ib*_; upper right panel). The spatial effect of inhibition is calculated as the point difference of the *E*_2_ and *E*_2*ib*_ surfaces (−*I*; lower left panel). The center-surround is represented as the difference the surfaces *E* and the *I* (lower right panel). The CPU time required to run one of the 20 simulations was 20 min on a Apple® MacBook Pro (Intel Core 2 Duo 2.93 GHz) plus 30 min to process the recorded data.

To compare our hybrid model with the biologically realistic model, we re-ran the simulations of Solinas et al. (2010b), using the code published in Solinas et al. (2010a). We changed the stimulus protocol to activate 13 MF terminals, instead of the original 8 terminals, to enlarge the stimulated area. This change brought an increase of the spot size to 33 μ*m* at 70% of the maximum peak amplitude, consistent with the published experimental evidence (Mapelli et al., 2010b). The network size was left at 100 μm by 100 μm as increasing it to the 200 μm by 200 μm size of our hybrid network model would imply a prohibitive computational cost. The recorded data were processed using the same procedure applied to the hybrid model. Figure 6 shows the stimulus effect 4.5 ms after onset, *E* peak, and at 8 ms, *E*_2_ peak. Like in the hybrid model the block of inhibition induces an increase of the network activity clearly visible at the time of the second peak. In the lower right panel of Figure 6 the spatial organization of the inhibitory feedback generates deeper spots in proximity of the central area, shaping the center-surround.

**Figure 7:**
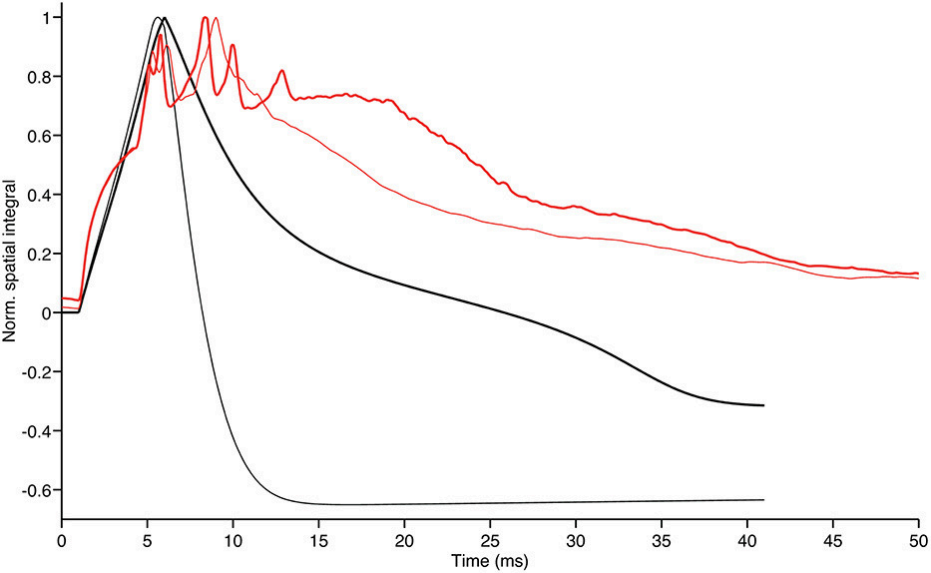
**Evolution in time of the GrC membrane potential shown in the upper left panels of Figures 5, 6 (thin black and thin red trace, respectively)**. The thick red and black traces show the time evolution of the same integrals after total block of inhibition in the 2010 model and partial block of inhibition in the hybrid model, respectively. Note that the thick traces generated by models with reduced inhibitory feedback have the tendency to stay above the thinner traces of model with control configuration as a result of more intense ensemble network activity in absence of inhibitory feedback. Furthermore, our hybrid model can follow the biologically realistic model up to the first peak *E* and conserve a qualitatively similar behavior at the time of the *E*_2_ peak.

To further highlight the similarities and discrepancies between the two GLN models we calculated the spatial integral of the activity surface for each time step of both models. The plot in Figure 7 shows how the time evolution of the hybrid model follows the traces of the biologically realistic model until the first peak is reached. From there on the discrepancy between the two models progressively increases but, in both models, the reduction of the inhibitory feedback leads to an increase of the overall model activity with the specific spatial distribution shown in Figures 5, 6.

Let us recall that our model constituted by (5)–(11) and (8)–(12) has been designed under strong simplifying assumptions that do not allow us to take into account the wide variety of phenomena in the single cell and in the whole network. Indeed, the FitzHugh-Nagumo model cannot reproduce the full complexity of the spiking activity produced by the Hodgkin-Huxley model. Furthermore, the GrC layer has been described as a continuum. Nonetheless, the remarkable result obtained is that our model is able to reproduce the benchmark dynamics present in Solinas et al. (2010b), at least in the time range in which the center-surround phenomenon arises. Concurrently, the delayed activation of GoCs allows the response of GrCs to the stimuli to survive till the GoCs inhibition arises. This configures a time window where GrCs are allowed to transfer their activity to the subsequent network layers. The intervention of GoCs inhibition closes this window resetting the GrCs activity and making them ready to reliably transmit a new stimulus.

**Figure 8:**
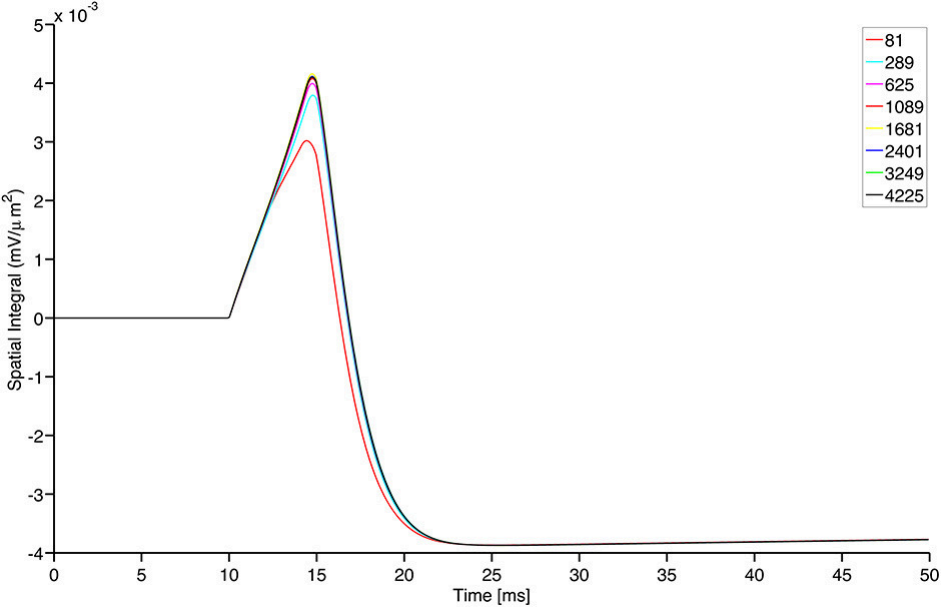
**Grid convergence for different structured grid resolutions of the continuous model for GrCs nodes**. The total number of nodes is shown in the plot legend. The plot shows the sum of the GrC membrane potential ω over the entire surface shown in upper left panel of Figure 5, divided by the area of the grid pixel and plotted as a function of time. Notably, only the coarser grid resolution shows a visible difference from the other resolutions and the difference among the solutions reduces for prolonged time intervals.

Finally, we conclude the present section by stressing that the simulations provided in this paper turn out to be independent of the GrCs continuous population grid refinement. Indeed, focusing on the framework that describes the center-surround phenomenon, we exhibit a comparison among the solutions produced by the model with increasing number of nodes in the space discretization of the GrC population. In Figure 8, we show the evolution in time of the integral of the activity over the network domain for different values of the spatial resolution. In practice, all the grid refinements we checked are able to catch the correct dynamics with sufficient accuracy. Convergence is clearly documented, thereby providing a sound background to the use of our numerical simulator.

### 3.3. Computational comparison

The computational performance of our new modeling method was assessed by running a simulation with an equivalent representation of a portion of the GLN in both simulators: NEURON (Carnevale and Hines, 2006) and our hybrid model simulator. The simulation used as reference is the one reproducing the center-surround effect in Solinas et al. (2010b).

On the one hand, in Solinas et al. (2010b) the full model simulation was run using NEURON to reproduce 420 ms of network activity (simulation run using the code available at Solinas et al., 2010a) and required 1080 s on a Apple MacBook Pro (Intel Core 2 Duo 2.93 GHz) for a network of 4001 GrCs and 27 GoCs and their synapses. On the other hand, considering an equivalent of 420 ms of activity with 4096 GrC vertices and 26 GoCs, the network we ran using our hybrid model simulator required 20 s (i.e., 47.6 times slower than a real time simulator). Therefore, our hybrid model simulator is roughly 54 times faster than the NEURON simulator. However, in our large network simulations we approximate the dynamics of small GrC clusters (composed of 13 GrCs) with a single node of the regular grid. Using the same approximation, we ran a simulation of the hybrid model with 361 nodes, 19^2^ nodes about 4096∕13. This simulation took 4 s to run, i.e., 270 times faster than the NEURON model (9.5 times slower than a real time simulator). We must also recall that the output of our simulator is immediately available for visualization in MATLAB while the output generated by the NEURON simulator requires an additional 30 min of post processing to be visualized. The compactness of our simulation setup allows running long and heavy simulations on a simple laptop as it was done for the simulation run to show the oscillatory network dynamics that reproduced the activity of 300, 000 GrCs for 1000 s. This simulation took 1.327 s and its corresponding NEURON network cannot be run on a single CPU computer. Moreover, our simulation can be interrupted, fully stored, and restarted at any time and it allows changing the configuration of the input and the network structure of the stored simulation before restarting it. The simulation can be run till a certain network state is achieved and multiple simulations can be started from the same network state but with different configuration of inputs.

This analysis quantitatively confirms the reduced computational cost of employing our simplified model instead of a detailed one, without losing information about such fundamental activity in time and space as the center-surround and the time-windowing. Let us stress that further improvements of our codes will lead to further time simulation savings. The most significant one will consist in translating our routines into parallel codes allowing us to take advantage of computer clusters or Graphics Processor Units (GPUs).

## 4. Discussion

With the aim of efficiently describing the dynamics of neuronal populations having a large density difference in specific brain areas, the present work collects new results next to the ones presented in Canuto and Cattani (2014). We started by stating the discrete conductance-based model (1) which describes the single cell membrane potential variation in time due to both electrical and chemical synapses. Afterwards, by letting the number of neurons tend to infinity, we derived the continuum model (4). The discrete and continuous models were then coupled to describe populations exhibiting significant differences in their densities, allowing us to formalize the hybrid model. Specifically, each cell of the low-density population was modeled by the discrete model, whereas the whole high-density population was described by the continuum model. Communications among populations, which translate into interactions among the discrete and the continuous models, are the essence of the hybrid model we presented. Such an approach, which leads to a significant computational cost reduction, was applied to the Golgi-Granular network in the cerebellum. Interesting dynamics such as microcircuit synchronization, traveling waves, center-surround and time-windowing were reproduced by the hybrid model. The two latter dynamics were compared with recent results in literature devoted to this specific network, confirming the capability of our approach to reproduce significant dynamics.

In this work we adopted a large number of approximations, explained in detail in Methods and Results. Nonetheless, our network model was able to reproduce salient features of the cerebellum GLN ensemble dynamics. However, this achievement is not surprising as the features the hybrid model can reproduce are strictly network emergent properties. The specific choice of neuronal or synaptic models is reflected in the exact timing of the first peak of the network response that we could approximate using the FitzHugh-Nagumo model.

It is interesting to highlight that the traveling wave phenomenon, which was not shown in the small network size and the short simulation time used in Solinas et al. (2010b), is not supported by evidence from experimental neuroscience. These traveling waves are of different origin from those induced by Purkinje cell recurrent collaterals in the developing cerebellum (Watt et al., 2009) and take origin from a network interaction limited within the GLN. Conversely, theta oscillations emerging in the hippocampus in freely behaving rats are present in the form of traveling waves (Lubenov and Siapas, 2009). However, based on the experimental data currently available on GLN neurodynamics, we can only conclude that our hybrid model is missing some features of the biological network that are fundamental to prevent the emergence of the traveling waves. Our first guesses to identify these missing features point to the simplified synaptic models currently present in our network model: the absence of the GoC-GoC inhibitory connections (Hull and Regehr, 2012) or the lack of inhibitory effects of the glutamate neurotransmitter on GoCs through metabotropic receptors (Watanabe and Nakanishi, 2003). These issues will be addressed in further computational work.

We wish to underline that the network size simulated here (1500 and 500 μm) is only one order of magnitude smaller than the rat cerebellar cortex (33, 000 and 6000 μm; Sultan and Heck, 2003). We expect that further improvements of the simulation speed, as code optimization for multi-thread processing and native code generation for the CPU and GPU hardware, may allow realtime simulations of full network size on ordinary computers.

In principle, these improvements should allow the simulation speed to reach the same levels of other software simulators, like EDLUT (Ros et al., 2006), NEST (Gewaltig and M., 2007), BRIAN (Goodman and Brette, 2009), and the hardware simulator SPINNAKER (Furber et al., 2013). Among these the EDLUT simulator can be used for a direct comparison as it was used to build a model of the GLN. EDLUT is able to run simulations of 100, 000 integrate-and-fire neurons in real time (Ros et al., 2006), as long as the overall network activity remains low, i.e., the total number of spikes traveling in the network is small. The same kind of limitation is affecting all the aforementioned simulators. Conversely, our hybrid simulator does not suffer from this limitation. However, it is yet unable to run real time simulations. Its inherently scalable mathematical basis, that allows to run a direct translation on massively parallel GPU hardware or FPGA, gives us confidence gaining simulation speed by applying simple changes to the original code.

By proceeding on the path here traced, some improvements should be taken into account in a forthcoming work. A major objective should concern how much the network behaviors here reproduced are related to the specific properties of the FitzHugh-Nagumo single-cell description. Moreover, one should evaluate if a different single-cell model is able to reproduce other significant behaviors such as resonant dynamics (Gandolfi et al., 2013). An extension to 3D representation of network structure can also be provided. This extension would find a direct application to the modeling of the cerebellum GLN as anticipated by anatomical studies of MF projections in this layer (cf. Figure 7 of Sultan and Heck, 2003). Finally, in order to make the model more realistic, future work should include the plasticity in communication strength among neurons.

Recent development of neuromorphic software highlighted the relevance of the interaction of sub-networks to build complex, high-level cognitive phenomena for processing and learning of verbal information (Golosio et al., 2015) or multilayered artificial neural networks able to perform very efficient learning representations of big datasets with multiple levels of abstraction (LeCun et al., 2015). In these examples the single neuronal units can be, and often are, a very simple abstraction of real neurons, far from biologically realistic neuronal models. Nonetheless, the whole network is able to perform surprisingly well or even outperform human operators in complex tasks. We believe that reduced models of interaction among sub-families of cells might be integrated to form composite models describing more complex brain activities. From this perspective, there is a precise interest in developing, testing and validating reduced models, oriented toward network level interactions — as done in the present paper for one specific kind of interaction.

## Author contributions

Part of this work has been done by AC during the Ph.D. program at the Polytechnic Institute of Turin under the supervision of CC. AC, SS, and CC contributed equally to design the study. AC and SS performed the study through numerical simulations. AC, SS, and CC interpreted the results. AC, SS, and CC wrote the paper. All authors have read and approved the final manuscript.

### Conflict of interest statement

The authors declare that the research was conducted in the absence of any commercial or financial relationships that could be construed as a potential conflict of interest.
